# Documenting the Provision of Emergency Contraceptive Pills Through Youth-Serving Delivery Channels: Exploratory Mixed Methods Research on Malawi’s Emergency Contraception Strategy

**DOI:** 10.9745/GHSP-D-24-00076

**Published:** 2024-10-29

**Authors:** Holly M. Burke, Philip Mkandawire, Mary Mulombe Phiri, Fannie Kachale, Kristen Little, Caroline Bakasa, Luwiza Puleni, Eden Demise, Paola Letona, Gwyneth Austin, Moses Kumwenda

**Affiliations:** a FHI 360, Durham, NC, USA.; b Family Health Services, Lilongwe, Malawi.; c Reproductive Health Directorate, Ministry of Health, Lilongwe, Malawi.; d Population Services International, Washington, DC, USA.; e Population Services International, Lilongwe, Malawi.; f Population Services International Latin America, Guatemala City, Guatemala.; g The LIFE Foundation, Blantyre, Malawi.

## Abstract

**Introduction:**

Emergency contraceptive pills (ECPs) are effective and can be used safely at any age repeatedly within the same cycle. They are often favored by youth yet are underutilized. Private facilities can increase ECP access but present barriers including cost. Identifying effective public-sector ECP distribution models can help ensure equitable access. The Malawi Ministry of Health developed a strategy to improve ECP access in 2020. We documented ECP provision through select public, youth-serving channels recommended by the strategy: general and youth-specific outreach, paid and unpaid community health workers (CHWs), and youth clubs.

**Methods:**

We conducted this mixed methods study from November 2022–March 2023 in 2 rural districts (Mchinji and Phalombe) implementing the strategy. We conducted qualitative interviews with 10 national stakeholders, 46 providers, and 24 clients aged 15–24 years about ECP service delivery. Additionally, 25 providers collected quantitative tally data about clients seeking ECPs. We analyzed qualitative data using grounded theory and quantitative data descriptively.

**Results:**

Stakeholders and providers reported ECP uptake increased in geographies where the strategy was implemented, especially among youth. Providers documented 3,988 client visits for ECPs over 3 months. Of these visits, 26% were from male clients, 36% were from clients aged younger than 20 years, and 64% received ECPs for the first time. Across channels, youth club leaders and unpaid CHWs reported the most client visits per provider and served the youngest clients. However, no ECPs were dispensed during 29% of visits due to stock-outs. While many providers were supportive of youth accessing ECPs, most held unfavorable attitudes toward repeat use.

**Conclusion:**

ECP access should be expanded through provision in the studied channels, especially youth clubs and CHWs. However, to meet demand, the supply chain must be strengthened. We recommend addressing providers’ attitudes about repeat use to ensure informed method choice.

## Abstract (precis)

Emergency contraceptive pill (ECP) uptake may increase for young people, first-time users, and those living in rural areas of Malawi by offering the method through public, youth-serving channels, especially youth clubs and community health workers. A national strategy focused specifically on this product can help grow ECP demand; however, the supply chain for ECPs must be strengthened to meet the additional demand.

## Teaser

**Key Findings:**

- Providers and key informants reported that emergency contraceptive pill (ECP) uptake increased where the strategy was implemented, especially among youth and in rural areas, but stock-outs hampered uptake.
- Youth club leaders and volunteer community health workers reported the most client visits per provider and served the youngest clients.
- Most providers had unfavorable attitudes toward using ECPs more than once per month or as a regular contraceptive method.

**Key Implications:**

- Policymakers in Malawi should expand their emergency contraception strategy to other parts of the country and improve the supply chain for ECPs.
- Program managers should build the capacity of youth clubs and community health workers to provide ECPs to reach young people and support providers to shift attitudes on repeat ECPs use.

## INTRODUCTION

### Emergency Contraceptive Pills

Emergency contraceptive pills (ECPs), distributed as 1.5 mg levonorgestrel (LNG) in a single pill or in 2 pills of 0.75 mg each, are safe and effective in preventing pregnancy after unprotected sex, including method failure, when taken within 120 hours (5 days).^1^ While they are more effective the sooner they are used after unprotected sex, studies demonstrated ECPs with LNG have a pregnancy rate of 1.2%–2.1%.^2^ ECPs work by delaying or preventing ovulation. ECPs do not cause an ectopic pregnancy and do not prevent implantation of a fertilized egg into the uterus.^3^^–^^5^ They do not induce an abortion if the woman is already pregnant. The World Health Organization (WHO)’s Medical Eligibility Criteria states that there are no medication restrictions for using ECPs, including age, and they can be used repeatedly within the same cycle.^6^ According to the WHO’s Selected Practice Recommendations, women should be given an advanced supply of ECPs so that they are available when needed and can be taken as soon as possible after unprotected sex.^7^ Research has shown that women are more likely to use ECPs after unprotected sex if they are given an advanced supply. Further, having advanced supply has not been shown to affect contraceptive use patterns or increase the use of ECPs or unprotected sex. ECPs do not protect against sexually transmitted infections, including HIV.

ECPs are a critical part of pregnancy prevention. They are desirable for those who have infrequent sex, those aiming to avoid longer-term hormonal side effects, those who are unable to afford or comply with the regimen of other contraceptive alternatives, or those who prefer a self-care method. They can be used safely as a primary, on-demand form of contraception, which evidence demonstrates is valued by some users and especially by those who may be unable to plan for sexual encounters, including adolescents and young women.^8^^,^^9^ Access to ECPs also presents an opportunity to “bridge” clients onto a regular and more effective contraceptive method if desired by the user, as well as to other sexual and reproductive health (SRH) services.^10^ Despite being less effective than other methods, individuals’ choice is paramount and should be respected if they decide to use ECPs. Indeed, family planning (FP) services should take a client-centered approach that responds to individuals’ preferences, needs, and values by honoring clients’ choice of method.^11^^,^^12^

Access to ECPs also presents an opportunity to “bridge” clients onto a regular and more effective contraceptive method if desired by the user.

The WHO strongly recommends that ECPs be routinely included within all national FP programs, be made available over-the-counter without a prescription, and be included in national guidelines on self-care interventions for health and well-being.^2^^,^^3^^,^^13^ In many countries, ECPs have gone from a prescription to an over-the-counter product, with private-sector pharmacies being a key driver of ECP access.^14^^–^^18^ Despite this shift, analysis of Performance Monitoring and Accountability 2020 data from across 8 African countries showed that less than 1% of all women had used ECPs within 12 months of the survey.^17^ Across studies, ECP users tend to be young (younger than 30 years), educated, employed, and unmarried.^18^^–^^22^ Further, ECP users are more likely to reside in urban areas, with the odds of using ECPs generally increasing with wealth.^19^^,^^21^ Known barriers to ECP access include facility hours not conducive to when the product is needed (i.e., nights or weekends), lack of knowledge, misinformation among end users and providers (e.g., that ECPs are a form of abortion), stock-outs, the perception that ECPs are being abused if used repeatedly, and stigma.^15^^,^^16^^,^^18^^–^^30^ Beyond pharmacies, which are primarily located in urban areas, there is limited information on specific service delivery channels in low- and middle-income countries that could facilitate increased ECP access.

### Emergency Contraceptive Pills in Malawi

Malawi has made great strides in increasing access to contraception. The country’s current modern contraceptive prevalence rate is 64.7% for women married or in union and 44.4% for sexually active unmarried women aged 15–49 years.^31^ Despite supportive policies, such as the National Youth Friendly Health Services Strategy,^32^ National Sexual and Reproductive Health and Rights Policy,^33^ and Adolescent Girls and Young Women Strategy,^34^ unmet need for FP remains at 44.8% for unmarried/sexually active women and is the highest among younger women ages 15–19 years at 71.5%.^31^

The public sector, where contraceptive products are offered for free, is the main source for contraceptive methods in Malawi.^35^ While ECPs were deregulated from prescription to over-the-counter status in 2015, data on ECP use in Malawi are limited. The Multiple Indicator Cluster Survey and Demographic and Health Survey reports include ECP use within the catch-all category of “other” methods. One small study among 419 pregnant women attending 3 antenatal clinics in southern Malawi in 2017 found that 2% had previously used ECPs.^36^ More recent data suggest ECPs may have been important in protecting some young women against pregnancy during the COVID-19 pandemic. Among modern contraceptive users responding to a phone survey (the majority of whom were aged 18–24 years), 4% were ECP users after the COVID-19 pandemic and 2% were ECP users before the pandemic.^37^ Stocks and use of ECPs in Malawi’s private sector is high (unpublished data). Most ECPs distributed in the public and private sectors are WHO prequalified and procured by the U.S. Agency for International Development and the United Nations Population Fund.^38^

### Malawi’s Emergency Contraception Strategy

Recognizing the importance of ECPs in Malawi’s method mix, the Reproductive Health Directorate of the Ministry of Health (MOH) developed a national emergency contraception (EC) strategy in 2020 (unpublished) to improve access and expand method choice among underserved populations, particularly youth, and in predominantly rural areas (where 83% of the population reside) through previously underutilized public-sector channels.^39^ These channels include several cadres of paid and unpaid community health workers (CHWs), youth clubs, and mobile outreach models focused on youth and adolescents. While the channels themselves were active in delivering a variety of FP-related services before the development of the EC strategy, none of these models included ECP distribution before the strategy launch. The strategy notes that these channels are in addition to the usual places where ECPs are found, such as victim support hotlines, colleges, and private-sector pharmacies and drug stores.

The MOH developed a national EC strategy to improve access and expand method choice among underserved populations, particularly youth, and in predominantly rural areas through previously underutilized public-sector channels.

The strategy outlines how to increase access to quality ECP information and services and expand access to ECPs through both private (for profit and nonprofit) and public sectors (including community-level distribution), with informed choice being a central component, in the following ways.

- Ensure adequate supplies to drive down prices in the private sector while increasing public service delivery points where ECPs can be obtained for free.
- Support service providers to ensure the National FP Program benefits from the ECP as a bridging product.
- Provide adequate information to users on the availability, safety, and use of ECPs, as well as have key opinion leaders provide community support to demystify ECPs and promote their safe use.

The strategy outlines who can use ECPs, when to provide ECPs, and who should dispense ECPs. The strategy specifies that there is to be no restriction on repeated use of ECPs and that they can be used repeatedly within the same cycle. This is a significant change, making Malawi a leader in the ECP space, especially within the context of informed choice and the advancement of SRH self-care interventions. The strategy also states that ECPs should be readily available; all women, girls, and men, regardless of age, should be informed about ECPs and dispensed ECPs in advance of unprotected sex; and women and girls can decide on their own whether ECPs are needed.

In 2021, MOH began implementing the strategy in a few districts by leveraging existing partners’ program platforms to accelerate implementation. To date, there has been no formal assessment of how Malawi’s strategy is shaping ECP service delivery through the novel delivery channels it proposes, particularly those that strive to meet the specific needs of youth. This study documented the provision of ECPs through the public sector, youth-serving channels recommended by the strategy. The findings will provide Malawian stakeholders with evidence to inform the scale-up of their strategy and add to the global body of evidence on approaches for expanding access to ECPs and method choice, including other contraceptive self-care options among young people.

## METHODS

The objectives of this exploratory, mixed methods study were to describe (1) how the national EC strategy has been implemented; (2) providers’ and program managers’ perspectives on implementation-related barriers and facilitators to ECP provision in select channels; and (3) who is accessing ECPs, how ECPs are being used (e.g., in advance of need, number of packs dispensed), and what are clients’ experiences (including barriers) accessing ECPs in select channels.

To maximize learning during the study, 5 channels were purposively selected, with the MOH and focused on public sector and youth-serving channels that provided the highest volume of ECPs (Box). In late 2022, when the study was designed, we conducted a mapping exercise to examine ECP distribution volumes by partner and channel over the preceding year. Population Services International (PSI) had the highest distribution volumes across the partners explored and had active ECP programs implementing the national strategy. During the period under review, PSI’s Tsogolo Langa project distributed the highest numbers of ECPs through mobile outreach (general population and youth-specific outreach), youth clubs, and the 2 CHW cadres in Malawi—paid health surveillance assistants (HSAs) and volunteer community-based distribution agents (CBDAs).

BOX Youth-Serving Channels Through Which Emergency Contraceptive Pills Were Distributed in Malawi

**General population outreach:** Population Services International (PSI)-owned mobile outreach program that offers family planning (FP) services for free at demarcated sites where a tent is set up every 2–3 months after demand is created in the communities.

**Youth-specific outreach:** Similar to general population outreach and shares the same staff members, but services are tailored to individuals ages 15–24 years, people who are single/not married, or those who never had a child. The key difference from generational population outreach is that this outlet is tailored to youth and the youth advise the mobile teams where to set up the tents.

**Health surveillance assistants (HSAs):** Paid community health worker cadre employed by the Ministry of Health (MOH) to provide FP services, including injectable contraceptives, since 2008, and since the adoption of the strategy, they now provide ECPs.

**Community-based distribution agents (CBDAs):** Volunteer community health worker cadre under MOH to provide FP services, including oral contraceptive pills, since the early 1990s, and since adoption of the strategy, they now provide ECPs.

**Youth clubs:** Government structures established and supported by the Ministry of Youth and the Malawi National Youth Council where youth have weekly activities related to health (sexual education, FP product distribution, and HIV) or daily income-generation activities. PSI layers ECP information, product, and services onto preexisting club activities. Trained youth club leaders are responsible for storing and distributing ECPs to their peers.

Two districts (Mchinji and Phalombe) implementing the strategy through the Tsogolo Langa project were selected for the study. While both districts are rural, Mchinji is in the central region of the country and Phalombe is in the southern region. These districts were selected because they were the longest-running programs and, therefore, had the highest volume of ECP distribution. The study was conducted in 3 traditional authority areas within the 2 districts from November 2022 through March 2023. We selected the areas with the most ECP providers trained across the selected channels. Through the Tsogolo Langa project, PSI trained the providers on ECPs during a 1-day training and distributed ECPs to the providers through a parallel supply chain system.

The study used a mixed method design, employing qualitative and quantitative research methods to examine the supply and demand sides of ECP service provision. For the supply side, we conducted 10 key informant (KI) interviews with national-level policymakers and ECP implementers to describe the ECP landscape and how it has changed since the strategy was launched (objective 1). KIs were purposively selected by the study team because they were either involved in developing the strategy or held a key position at an organization implementing the strategy. We also conducted semistructured interviews with 46 ECP providers and program managers sampled from the 5 selected channels (approximately 6 per channel) in Mchinji and Phalombe districts to hear their perspectives on how ECPs are being provided and identify implementation-related barriers and facilitators to providing ECPs in their channels (objective 2). Providers were purposively selected with support from MOH leaders if they had provided (or supported a program that provided) ECPs to at least 1 client in the past 6 months. The same providers cover both general and youth outreach events, so there were only 4 cadres of providers included in this part of the research. Outreach events are covered by 1 team per district.

For the demand side, we conducted in-depth interviews with 24 ECP clients sampled from the channels to describe clients’ experiences accessing ECPs through the various channels (objective 3). The demand-side research was conducted only in Mchinji District. Providers referred interested clients ages 15–24 years who received ECPs from the channels within the month before the interview to study staff until the desired sample size of approximately 6 interviews per channel was reached. Additionally, 25 ECP providers in Mchinji district used paper tally sheets to collect brief, nonidentifiable quantitative data (sociodemographic characteristics and how clients were using ECPs) about clients seeking ECPs for 3 consecutive months in the select channels (objective 3). As it turned out, there were no youth outreach events held during this data collection period. All eligible outreach (n=7) and youth club (n=5) providers in the study areas collected data. However, there were too many CBDAs and HSAs to feasibly include in the study, so program managers purposively selected those who provided the most ECPs in the past 12 months. Of the 30 CBDAs and 17 HSAs who were eligible to be included, 8 and 5 collected data, respectively. All participants provided informed consent before being interviewed.

The targeted sample sizes were based on the resources available and previous research by Guest et al.^40^ that showed that 6 interviews would be sufficient to develop overarching themes and useful interpretations when using specific domains of inquiry, studying a relatively homogenous group, and aiming to understand common perceptions and experiences. All interviews were conducted by experienced and trained researchers in the participants’ preferred language (English or Chichewa) and audio-recorded with participants’ permission. Verbatim transcripts were prepared in English. Quantitative data were entered from paper forms into electronic Open Data Kit (getodk.org) databases.

For the qualitative data, we conducted a separate thematic analysis of the transcripts from the 3 study populations using grounded theory.^41^^,^^42^ The study team developed 3 separate codebooks that combined a priori codes for key concepts related to the study objectives with data-driven codes identified through initial reading of the transcripts. We used Dedoose qualitative software (version 9.0.17) to organize the data for analysis. Coding was done by 3 analysts. Intercoder agreement was assessed on 10% of all transcripts until high consistency was achieved. We then examined codes for subthemes and patterns across the transcripts. We summarized the information by major themes in 3 memos. Quantitative data were summarized descriptively: demographic data were collected from interview participants using Microsoft Excel and tally sheet data using STATA statistical software (version 17, Stata Corp, College Station, TX).

### Ethical Approval

The study was reviewed and approved by the National Committee on Research in the Social Sciences and Humanities in Malawi (Protocol number: P.07/22/661) and FHI 360’s Protection of Human Subjects Committee in the United States (Project number: 1909937-1). The tally sheet data are not defined as human subjects’ research because they did not identify any person specifically, and, therefore, consent was not sought for this activity.

## RESULTS

### Interviewee Characteristics

All 10 KIs were based in Malawi and worked for a variety of organizations: 5 local organizations, 2 international organizations, 2 members of MOH, and 1 from a donor agency (Table 1). KIs averaged more than 10 years of experience. Semistructured interview participants (n=46) were overwhelmingly involved in direct delivery of ECPs (n=43); thus, we refer to them here as providers. Providers’ representation across the districts and channels were similar, except there were fewer from youth clubs (n=9). Providers had a mean age of 32 years and were mostly male (67%).

**TABLE 1.** Sample Size by Type of Data Collection, Malawi

	**No.**			
	**Key Informant Interviews**	**Provider Semistructured Interviews**	**Client In-Depth Interviews**	**Tally Sheet Data Collection, Providers Engaged**
**National**				
Local organizations	5			
International NGO	2			
Ministry of Health	2			
Donor	1			
**Mchinji district**				
Youth outreach	--	6	4	7
General outreach	--		6	
HSAs	--	6	1	5
CBDAs	--	7	7	8
Youth clubs	--	3	6	5
**Phalombe district**				
Youth outreach	--	7	--	--
General outreach	--		--	
HSAs	--	6	--	--
CBDAs	--	5	--	--
Youth clubs	--	6	--	--
**Total **	10	46	24	25

Abbreviations: CBDA, community-based distribution agent; HSA, health surveillance assistant; NGO, nongovernmental organization.

Of the 24 ECP clients we interviewed, 75% (n=18) were female, 75% (n=18) were aged 18–24 years, and 75% (n=18) were living with their family, 92% (n=22) were unmarried, and (71%) (n=7) had a child. The majority (58%) (n=14) completed only primary education. Clients reported last receiving ECPs from a CBDA (n=7), general outreach (n=6), youth clubs (n=6), youth outreach (n=4), and an HSA (n=1). Due to training and implementation delays, HSA distribution had not yet been scaled widely at the time of the interviews.

Most clients we interviewed reported ever using short-term contraceptive methods (20 of 22 clients asked this question), while 2 clients reported ever using implants as well as short-term methods. Among the 20 clients who have ever used short-term methods, most (n=17) reported having used condoms, and a few used injections (n=3) or oral contraceptive pills (n=2). Among the 17 clients who had used condoms, all reported having used them to prevent pregnancy (n=17), and/or most used them to prevent sexually transmitted infections (STIs) (n=15). There were no large differences in clients’ previous method use and channel last visited for ECPs. Half of the clients (n=12) reported currently using condoms along or interchangeably with ECPs.

*We use emergency contraceptive pills, but when they run out, like it is now, we use condoms.* —CBDA client, female, age 18–24 years

### Perspectives on Emergency Contraception Strategy Implementation

All but 1 KI (n=9) knew about the strategy before the interview, and those who were aware felt the strategy was working well overall. They said that many organizations were involved in developing the strategy and felt that the strategy had increased the focus on youth and rural areas and had increased ECP uptake. They mentioned these areas for improvement: further disseminating the strategy because some districts had not been reached yet, continuing to increase ECP demand, improving the supply chain, and having a better supply plan. KIs reported several challenges to implementing the strategy, including poor management and quality of FP data and the inability to link private-sector data with those from the public sector. Quantification of ECP commodities was also mentioned, with informants noting that poor data often led to inaccurate forecasting, resulting in stock-outs or stock levels that were not aligned with demand. The length of time it took to get budgets approved was another challenge reported. Several KIs noted that the supply of ECPs could not meet current demand and called for a better supply plan.

KIs felt that the ECP strategy had increased the focus on youth and rural areas and had increased ECP uptake.

*The recommendation is to have enough stock …ensuring that the stock management leadership at the facility, hospital, clinic or whatever, are well aware that it’s one of the methods that’s most preferred by young people among others.* —KI, international nongovernmental organization, female, age 28 years

Fewer than half of the 46 participating providers (n=18) were aware of the strategy, and 1 respondent noted that awareness was constrained by the limited geographical roll-out of the strategy to date.

*Yes. I wish that things about the strategy should be disclosed to everyone so that people should know that this is being implemented at a national level. These things must be known by everybody because these services are important. From my understanding this [strategy] is not being implemented in all areas but only to few randomly selected areas. It could have been better if it was being implemented country wide.* —HSA provider, Mchinji, male, age 47 years

### Perspectives on Implementation Barriers and Facilitators

Across both districts, providers highlighted what had been going well with ECP services, and many shared more than 1 reason. Of the 43 providers who commented on this, most mentioned a positive impact of the strategy on the community, including the perception of fewer unplanned pregnancies (n=21) and increased school continuation (n=3).

*It must have been the past year when schools were suspended due to COVID when rumors circulated that in Phalombe district…where many young girls, about 5,000 had dropped out that time. More ECPs were distributed, which possibly assisted in preventing unwanted pregnancies among many young girls.* —Outreach provider, Phalombe, male, age 41 years

The second most common reason was the providers’ perception that knowledge about and awareness of ECPs improved because of different demand creation efforts (n=10), which they believed contributed to higher demand for ECPs (n=11). Providers also described positive client experiences through improved delivery of ECP services in the community and clients’ increased comfort seeking ECPs (n=8).

According to some providers, the most common challenge to delivering ECP services was around the health system having adequate ECP supply (n=37). When 44 providers were asked explicitly if ECP supply was meeting demand, most providers said “no” (n=38). They noted that demand for ECPs had been high for several reasons, including stock-outs of other methods.

Providers reported that the most common challenge to delivering ECP services was around the health system having adequate ECP supply.

*Okay, I can be given 20 packets, yet I have about 5 CBDAs including myself, if given 20 we divide 4 or 5 to have equal share, so if a client comes, I can’t give her/him 3 packets otherwise I will remain with only 1 packet. With this I would say we don’t have enough stock for the high demand. We are supposed to give the client 4 to 5 packets so that it should take time before they come back for other packets, and this is what we learnt during COVID-19 period so that we shouldn’t be frequently meeting people. —*CBDA provider, Phalombe, male, age 32 years

Some providers (n=6) reported that other providers still lacked knowledge and training around ECPs (n=6) or did not have adequate transport to deliver ECPs (n=5). Providers also reported challenges concerning clients. Some providers felt clients lacked accurate information about ECPs (n=5), had heard false rumors about ECPs (n=4), and feared judgment to obtain ECPs (n=2). By channel, those from the CBDA channel were more likely to report health system challenges, and those from the youth club channel were more likely to report provider-level challenges described earlier.

### Channel Reach and Client Characteristics

**FIGURE 1:**
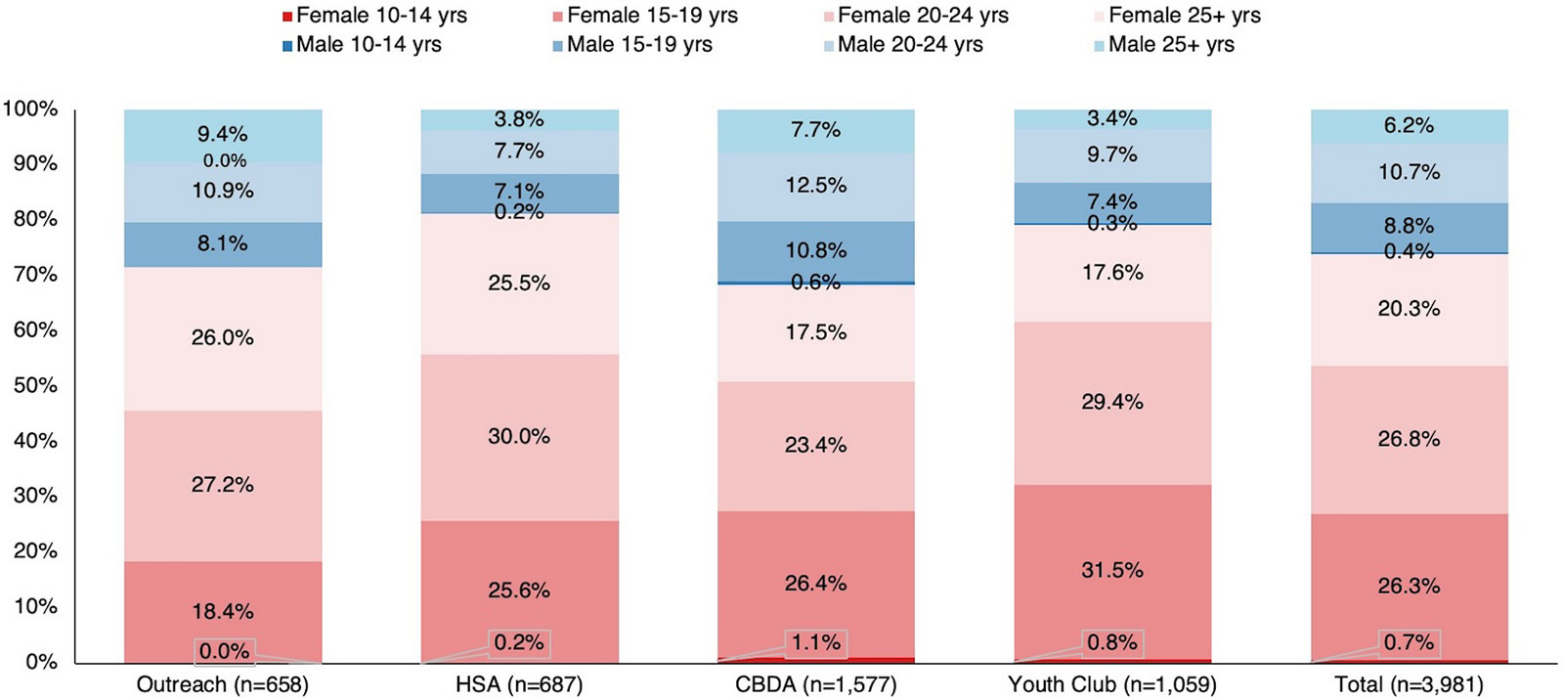
Percent Distribution of Age-Sex Category of Client Visits by Delivery Channel, January–March 2023, Mchinji District, Malawi Abbreviations: CBDA, community-based distribution agent; ECP, emergency contraceptive pill; HSA, health surveillance assistant.

From January through March 2023, 25 providers documented 3,988 visits from clients seeking ECPs (Table 2). The clients’ ages ranged from 13 to 56 years, with the average being 22 years. One percent of the total visits, or 40 visits, were made by clients ages 10–14 years (Figure 1). CBDA and youth club providers had the highest mean number of visits per provider, 198 and 140, respectively, whereas the outreach providers had the fewest at 65 visits. The lower outreach numbers may have resulted from the program just restarting at the time of data collection. Though differences were relatively small, youth clubs and CBDAs documented the youngest clients (average age: 22 years), and outreach documented the oldest clients (average age: 24 years). First-time ECP users made up 64% of visits. There was some variation by channel, but not substantial (range: 61% for HSAs to 68% for outreach). More than a quarter of ECP visits were made by male clients. While all channels served male clients, CDBAs and outreach documented the highest proportion of visits by male clients (32% and 28%, respectively, compared to 19% and 21% in HSA and youth clubs).

**TABLE 2.** ECP Client Visits by Delivery Channel, January–March 2023, Mchinji District, Malawi

	**General Outreach**	**HSA**	**CBDA**	**Youth Club**	**Total**
Providers, no.	7	5	8	5	25
Client visits, no.	659	686	1,581	1,062	3,988
Visits per provider, mean, no.	65	137	198	140	160
Percentage of total visits, %	17	17	40	27	100
Age, years, mean (range)	24 (15–49)	23 (14–56)	22 (13–51)	22 (14–45)	22 (13–56)
Visits by first time ECP users, no. (%)	447 (68)	419 (61)	998 (63)	667 (63)	2,531 (64)
Visits by female clients, no. (%)	471 (72)	558 (81)	1,078 (68)	839 (79)	2,946 (74)

Abbreviations: CBDA, community-based distribution agent; ECP, emergency contraceptive pill; HSA, health surveillance assistant.

Examining age-sex categories (Figure 1), we observed 32% of youth club client visits were made by girls ages 10–19 years (0.8% were made by girls ages 10–14 years) and 29% by women ages 20–24 years. The CBDA channel had the highest proportion of visits (11%) by boys ages 10–19 years of age (0.6% were made by boys ages 10–14 years).

During the in-depth interviews, we asked ECP clients which channel they preferred to receive ECPs. Of 15 clients who responded, the most common responses were youth club (n=6), followed by CBDA (n=4) and hospital (n=2). Some of the reasons that clients gave for preferring youth clubs were that the ECP provider (referring to the youth club leader) was close by or within their neighborhood and because the provider usually had ECPs in stock. Some clients mentioned they felt less judgment from the youth club providers compared to hospital staff.

*Because the service provider lives within our neighborhood at [states neighborhood name], so when I ask him to take the ECP here and bring me home, he does exactly that.* —Youth club client, female, age 15–17 years

*[I prefer the youth club] because everyone opens up. We are able to ask questions that we can’t ask at the hospital.* —Outreach client, female, age 18–24 years

During the semistructured interviews, of the 29 providers who were asked if ECPs should only be discussed with those who asked for it, 25 providers said “no.” Most felt that ECPs should be discussed with those who did not ask for it so that people of reproductive age would be able to make informed decisions about different methods. One provider reported an unfavorable attitude about youth using ECPs because ECPs are not as effective as other methods.

*I feel good seeing the youth using ECPs, you know youth of nowadays started doing wrong things whilst young and this disrupts education, especially for the women. They get pregnant but with the use of pills things became good and they can continue with their education without any disruption.* —CBDA provider, Mchinji, male, age 26 years

*I prefer if they [youth] used other FP methods because if they use ECP they can get pregnant because they are not 100% effective. At least they should opt for a better method that can help them.* —Outreach provider, Mchinji, female, age 27 years

### How Emergency Contraceptive Pills Are Being Used

The providers collecting the tally sheet data were asked to document each visit by clients seeking ECPs over the observation period. However, for 29% of these visits, clients did not receive any ECPs primarily due to stock-outs (Table 3). HSAs and CBDAs had the highest proportion of client visits in which ECPs were not obtained (36% of visits), and outreach had the lowest proportion not served (12%). Among visits where ECPs were received, 31% received only 1 pack. The number of packs dispensed varied by channel. Youth club providers distributed 1 pack during 46% of their visits, whereas HSAs gave 3 or more packs during 56% of their visits. Outreach providers were more likely to distribute 6–10 packs, which they did for 20% of their visits, perhaps because they provided periodic services compared to the other channels.

**TABLE 3.** ECP Visits Where ECP Was Dispensed by Channel, January–March 2023, Mchinji District, Malawi

	**General Outreach**	**HSA**	**CBDA**	**Youth Club**	**Total**
Providers, no.	7	5	8	5	25
Visits where ECP was dispensed, no. (%)	577 (88%)	457 (67%)	1060 (67%)	745 (70%)	2,839 (71%)
No. packs distributed at visit, no. of visits (%)					
1	143 (25%)	39 (9%)	342 (32%)	341 (46%)	865 (31%)
2	209 (36%)	159 (35%)	358 (34%)	221 (30%)	947 (33%)
3–5	107 (19%)	215 (47%)	312 (29%)	178 (24%)	812 (29%)
6–10	116 (20%)	38 (8%)	41 (4%)	4 (1%)	199 (7%)
10 or more	2 (0%)	6 (1%)	7 (1%)	1 (0%)	16 (1%)

Abbreviations: CBDA, community-based distribution agent; ECP, emergency contraceptive pill; HSA, health surveillance assistant.

During the interviews, we asked the providers how many packs they usually distributed and most said 1–3 packs. Some providers noted that the number of packs varied by factors including availability, if a client was in boarding school, distance the client traveled, or if the client was using other methods.

*Usually it is only 1 pack [I give out]. Because they are scarce so if we give them more than 1, we can run out of stock.* —Outreach provider, Mchinji, female, age 27 years

Although providers tended to give out few packs, they said that most clients requested 5 or more packs.

*I wanted to receive 6 packets because these youth day events take months to come along, so it doesn’t happen often.* —Outreach client, female, age 15–17 years

**FIGURE 2:**
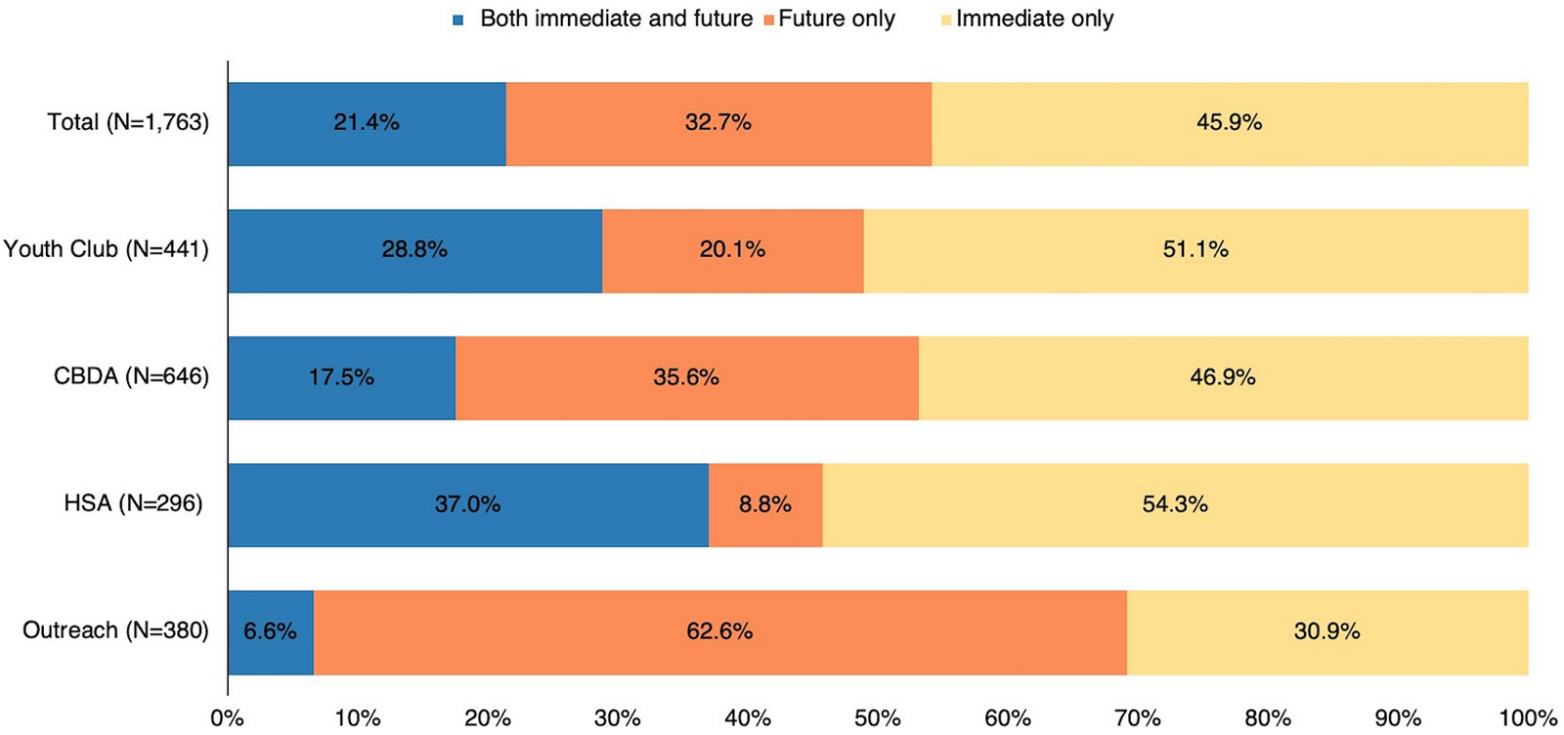
Among Visits Where ECP Was Received, Client Reported Timing of ECP Use by Delivery Channel, January–March 2023, Mchinji District, Malawi Abbreviations: CBDA, community-based distribution agent; ECP, emergency contraceptive pill; HSA, health surveillance assistant.

Providers also documented in the tally sheet whether ECPs were being obtained for immediate or future use, (or both, if multiple packs were dispensed). During 46% of the 1,763 visits, clients reported getting ECPs for immediate use only, while during 33% of the visits, clients reported it was for future use only (Figure 2). Outreach had the highest proportion of clients (63%) planning to use the ECPs in the future.

Many of those who obtained ECPs reported already using other contraceptive methods or planned to use another method (data not shown). Among nearly 70% of visits, the clients obtaining ECPs for immediate use reported that the ECP user was using another method (the person obtaining ECPs did not have to be the user but could be a friend or partner). This was reported most often during youth club visits (77%) and least often during outreach visits (56%). Overall, during more than half (58%) of visits, the clients who were getting ECPs for future use reported that they would use other types of methods with ECPs in the future. The highest was for CBDA (67%) and lowest for HSA visits (46%).

We used the information from the client interviews to help understand the quantitative findings from the tally data. The clients who participated in the interviews reported a variety of reasons why they obtained ECPs at their last visit. Of the 22 participants who answered this question, 11 specifically mentioned they obtained ECPs for future use. Other less common responses included: protection against pregnancy (n=9), had unprotected sex (n=9), obtained for someone else (n=3), and for extra protection (n=2).

Though we did not ask about it explicitly, misconceptions arose spontaneously. Two clients said they were using ECPs to protect against STIs.

*I did not want to have unexpected pregnancies. … I also did not want to get sexually transmitted diseases.* —HSA client, female, age 18–24 years

In addition to holding this same misconception, a male client from youth outreach incorrectly believed that ECPs can be taken by men and stated the outdated duration of ECPs (72 hours instead of 120 hours).

*Aah when it comes to contraceptive emergency pills then they are used by women, but I can just say yes, I can say yes, they can be used by anybody. Anybody who has them at that time. And if he drinks the pills within 72 hours, that means every disease whether gonorrhea, syphilis, genital herpes, HIV, that means the diseases will be inactive*. —Youth outreach client, male, age 18–24 years

Most of the clients interviewed reported learning about ECPs through youth clubs (n=14/23 who were asked). The second most cited source was interpersonal channels, including friends, network, or boyfriend (n=5), followed by CBDAs (n=3), and hospitals (n=2). During the interviews, we asked clients what the provider told them about ECPs (Table 4). The most common response among those asked (n=20/24) was restrictions or requirements (n=18), followed by how to use ECPs (n=12), efficacy (n=15), frequency of use/repeat use (n=9), when to use ECPs (n=9), how ECPs work (n=8), and side effects (n=6). There were not large differences in responses by channel. While most clients reported being told correct information, 2 responded to this question with incorrect information, including that ECPs protect STIs and the outdated duration for ECPs (72 hours instead of 120 hours):

**TABLE 4.** Clients’ Report of What Providers Told Them About ECP, From In-Depth Interviews

**Type of Information Provided by Provider** ^a^	**Total (n=20)**	**Stratification by Outlet Client Last Visited**				
		**CBDA**	**General Outreach**	**Youth Club**	**Youth Outreach**	**HSA**
Restrictions/requirements	18	5	5	4	3	1
Efficacy preventing pregnancy	15	3	5	4	2	1
How to use ECPs	12	2	2	3	4	1
Frequency/repeat use	9	3	1	3	1	1
When to use ECPs	9	3	3	3		
How ECP works	8		4		3	1
Side effects	6	1		4	1	
Dosage	3		1	1		1
ECPs do not provide STI protection (correct)	2				1	1
ECPs provide STI protection (incorrect)	1				1	
ECPs are for emergency use	1		1			
No long-term effects on fertility	1					1
Other pills (not further specified) to take along w/ ECP to protect against STIs	1			1		

Abbreviations: CBDA, community-based distribution agent; ECP, emergency contraceptive pill; HSA, health surveillance assistant; STI, sexually transmitted infection.

a Multiple responses possible.

Most of the clients interviewed reported learning about ECPs through youth clubs.

*They said you should be taking them when you are about to have sex and also when you want to prevent sexually transmitted diseases. That’s all.* —Youth club client, female, age 15–17 years

*They said they are to be taken by a person who has had unprotected sex. They are supposed to take them after 72 hours. I mean before 72 hours elapse.* —Youth outreach client, female, age 15–17 years

Among the 32 providers who commented on when ECPs should be taken, 28 said that ECPs should be taken within a 72-hour window, while only 4 said that ECPs should be taken within a 120-hour or 5-day window.

*For the ECP, if it is more than 72 hours after sex, the pills will not work.* —Outreach provider, Phalombe, male, age 37 years

*Aaaa what I know about EC strategy is that they added strength from 3 days or 72 hours but now it is 5 days for ECP to be effective that’s what I know changed.* —Youth club provider, Phalombe, male, age 22 years

During the interviews, we asked providers what they thought about clients using ECPs more than once in the same month. Among the 44 providers who answered this question, 20 had unfavorable attitudes. The most common reason (n=10) for having unfavorable attitudes was because providers felt that these clients should be using another method that was more effective. By channel, most of those with unfavorable attitudes were from the outreach channel, while most of those who had favorable attitudes were from the CBDA channel. We also asked 38 providers for their perspectives on clients using ECPs as a regular method, and 19 of these had unfavorable attitudes. By channel, the majority of those who had unfavorable attitudes were HSAs.

*…they need to be given more information on other [FP] methods because these are emergency pills, and it’s not the only way to prevent unplanned pregnancies. …They can have a wide variety of choices and…can know that these emergency pills are for emergency use, but there are more sustainable methods. So, we can tell them about condoms, or pills or the other methods.* —HSA provider, Phalombe, male, age 49 years

During the interviews, we asked providers about whether women and girls might be coerced into taking ECPs by their male partners. Of the 21 providers who discussed this topic, 5 said that women were and 1 said that women might be coerced into taking ECPs by their partners. However, most providers who were asked said they had not heard of clients being coerced.

### Provider and Client Perspectives on Improving Emergency Contraceptive Pills Services

Providers gave a variety of reasons as to how ECP services could be improved. The most reported opportunities concerned the health system and included improving the supply of ECPs (n=30/46), logistics (n=18/46), and data systems (n=13/46). The next common responses were about providers and included having trainings or refreshers on ECP service provision (n=36/46), additional supervision (n=12/46), and providing incentives to providers (n=9/46). Providers also said more awareness about ECP services was needed (n=35/46). By channel, similar proportions of providers from across the channels reported that health systems improvements were needed, while more CBDAs and HSAs reported improvements for providers.

The clients interviewed also had several recommendations for improving ECP services, most commonly concerning increasing the supply, consistency, and availability of ECPs (n=7/23).

*I think the supplies of the pills should be consistent, because sometimes we find that they have run out of stock when we go to get some.* —Youth club client, female, age 18–24 years

*Madam [provider] should have a lot of things so that we shouldn’t be suffering…. She should have ECPs, condoms, and other FP methods so that we just collect from her. Right now, we have to wait for another gathering so that we get ECPs.* —Outreach client, male, age 15–17 years

Clients also suggested increasing the number of ECP providers and having them closer to their homes (n=4/23).

*If they could have the service providers right in our communities whereby, they can be providing the ECPs door by door, it could really help.* —Youth club client, female, age 18–24 years

Related, some clients recommended increasing the number of outlets offering ECPa and specifically mentioned youth clubs, CBDAs, and outreaches (n=3/23).

*I think there is need for additional CBDAs so that if one doesn’t have (ECPs) the other may be able to have, and sometime there is too much work for them, they cover big villages and they get overwhelmed with walking long distances and they don’t have any means of transport. So, it is difficult for them to reach many people in a short time.* —CBDA client, female, age 18–24 years

Client participants also recommended sensitization activities to increase ECP awareness and demand (n=4/23). We asked clients about costs they incurred getting ECPs. Most said that they did not have to pay for ECP services; however, many experienced opportunity costs, such as missing work.

## DISCUSSION

Malawi’s strategy recommends offering ECPs in underutilized, public-sector distribution channels, including youth clubs, CBDAs, HSAs, general population outreach, and youth-specific outreach. This study explored the implementation of the strategy in 2 districts where it has been rolled out, including key stakeholder, provider, and client perspectives on ECP service delivery since the strategy was introduced, as well as opportunities for improvement.

### Implementation of the National Emergency Contraceptive Pill Strategy

During this study, KIs and providers described their perceptions of increased ECP uptake since the launch of the strategy. We documented thousands of ECP visits over 3 months, including by young people and first-time users, through the channels that we studied, especially youth clubs and CBDAs. These findings support stakeholder and provider perceptions that the national strategy had increased demand for and access to ECPs for clients in districts where it has been implemented, especially for youth and those in rural areas.

While having a standalone strategy for ECPs shone light on this underutilized method, in the long term, it may be more efficient to integrate ECPs into a broader strategy. Specifically, the demand for ECPs observed in this study suggests opportunities to build on Malawi’s successes in introducing self-care policies and programs by linking ECP service delivery to other offerings. As self-care gains wider recognition and policy support globally, including in Malawi, there may be further opportunities to support self-directed information provision and other SRH-focused self-care interventions, including ECPs. This may be accomplished by integrating ECPs into a community-level, adolescent-responsive self-care package.^43^ This package could include a range of self-care options tailored to the unique needs of adolescents and young people, such as HIV self-testing, pre-exposure prophylaxis, condoms, self-injectable contraceptives, self-directed information designed for and with young people, and linkages to further care and the formal health system. Because these interventions may require more limited interaction with the formal health system relative to provider-administered alternatives, identifying strategies for effective, efficient, and youth-friendly alternatives for counseling and accurate FP information provision only become more critical. Digital strategies using popular social media platforms, influencers, and user-responsive systems, like chatbots or AI, should also be considered.

The findings support stakeholder and provider perceptions that the national strategy had increased demand for and access to ECPs for clients in districts where it has been implemented, especially for youth and those in rural areas.

In addition to expanding the channels for ECPs, the strategy also set out to ensure there should be no restriction on repeated use of ECPs, which is especially important when ECPs are used as a standalone method. However, in this study, we found that ECPs were often used with other methods. Nevertheless, most providers we interviewed had unfavorable attitudes toward using ECPs more than once per month or as a regular, standalone method. Similar concerns and provider perceptions that ECP provision to youth and adolescents would encourage sexual activity or earlier sexual debut have been noted in previous studies.^14^^,^^44^ While some of the concerns highlighted in our study stemmed from a desire to see adolescents using more effective methods, ECPs were often described as being intended for “emergency” situations only. These perceptions may be influenced by the lack of supply, the product’s name, and global guidance that historically recommended ECPs as a short-term and transitional method rather than a standalone method.^45^ Given the critical role providers play in enabling ECP access and information provision, as well as shaping attitudes and perceptions of ECPs among end users, ensuring that providers have accurate information about ECPs and mitigating provider biases will be critical to successful scale-up of ECPs in Malawi and elsewhere.^46^ We recommend a heightened focus on increasing providers’ knowledge on the duration for ECPs, the safety of repeat use of ECPs, as well as deliberate efforts to ensure FP services are client-centered by addressing provider values and norms around adolescent sexuality and method choice. At the same time, social and behavioral communication change activities are needed to address misconceptions that continue to persist about how ECPs work and what they can and cannot do. Our study identified several areas where Malawi’s service delivery guidelines could be strengthened with more details about ECPs. For example, providing recommendations on the number of packs to dispense and encouraging more packs per need, counseling for other methods and condom use, especially for youth, and partnerships with the Ministry of Education to further expand access to ECPs and reduce school dropout due to unplanned pregnancy.

These efforts may have the added benefit of improving the enabling environment for the potential introduction of new contraceptive technologies, including on-demand contraceptive pills,^47^ which are intended to be taken pericoitally as a regular form of nonemergency contraceptive.^48^ Future research in Malawi is being planned to further explore end user preferences for on-demand contraceptive pills. However, the most salient finding across KIs, providers, and clients in our study is that while demand for ECPs was high, stock-outs and supply chain challenges hampered provision and uptake. Indeed, the rationing behavior that providers described by dispensing fewer ECP packs than clients requested is a response to larger, structural barriers that are operating across the health care system that disrupt method choice. Moreover, ECPs were not the only method that was stocked out during the study period. The lack of supply of other methods may further increase demand for ECPs as a backup option when preferred methods are out of stock. Supply chain challenges likely already impede choice for end users, which has been noted by providers in previous studies,^49^ and would be a key barrier to the introduction of new contraceptive technologies as well as further scaling of existing options like ECPs. Therefore, implementation of the national EC strategy can be most improved by reexamining the supply chain, especially for commodities that go to the community, and addressing bottlenecks that affect ECP supply. This includes improving stock management, forecasting, and monitoring data on ECP supply and uptake in both the public and private sectors.

### Reach of Novel Distribution Channels for Emergency Contraceptive Pills

We found that 4 novel ECP distribution channels (youth clubs, CBDAs, general population outreach, and youth-specific outreach) received nearly 4,000 visits from clients seeking ECPs in a single district over a period of 3 months. Nearly 54% of visits were made by women and girls ages 10–24 years, ranging from 45% of outreach visits to 60% of youth club client visits. Among the channels surveyed, youth clubs and HSAs were particularly effective at reaching young girls. While all channels reached a sizable number of boys and men, CBDAs stood out for having the highest proportion of male clients. Our qualitative data suggest that young people prefer channels that are closer to home and where they experience less stigma for using ECPs. Other research from the region, including Malawi, has suggested that while anticipated stigma can be a barrier to accessing ECPs for all women, this may be most significant for young women who fear being discriminated against or mistreated for being sexually active.^10^^,^^50^ Our findings also demonstrate the value of providing FP commodities such as ECPs through a diverse set of public sector channels that increase availability, choice, and access for potential end users.

We found that 4 novel ECP distribution channels received nearly 4,000 visits from clients seeking ECPs in a single district over a period of 3 months.

We could consider leveraging preferences for convenient, less stigmatizing ECP sources by providing more training on other methods to these preferred providers. This may serve to increase bridging to longer-acting or more effective methods if and when the client may be interested in learning more about other contraceptive options. PSI has developed a job aid about all methods for their mobilizers to use in communities—this job aid could be used with CHWs and youth club leaders in the future, but we must be cautious about not layering too much onto the volunteers’ scope of work. Further, we do not know if the job aid would be used regularly or if young people would be receptive to additional counseling from these ECP sources. However, given the instances of misinformation we heard during the interviews—a finding that has been noted in other studies looking at ECP knowledge and perceptions among women and youth^10^^,^^11^^,18^—identifying ways to provide high-quality counseling to youth will be important to ensure that they are able to use ECPs safely and effectively and to confidently access other methods when and if they need them. The high impact practice (HIP) enhancement for adolescent-responsive contraceptive services provides recommendations on how this may be accomplished (e.g., whole clinic training, low-dose and high-frequency trainings, complementary trainings that include values clarifications exercises).^43^

### Strengths and Limitations

This study has several limitations. The study included only 2 geographies, which were purposively selected to examine regions where the strategy has been implemented. We did not include a comparison geography or compare time periods before/after the strategy rolled out. Because we selected areas where the most ECP providers had been trained and the highest ECP volumes were distributed, we may have biased our findings toward geographies where implementation of the strategy was likely to be strongest. The tally sheet data may contain repeat clients because providers documented visits (rather than unique clients). We attempted to measure within provider dependence by asking the providers to indicate if this was the first time this person was recorded in their tally sheet and found that at least 34% of visits were from repeat clients. We say “at least” because we think the historical ECP shortages could have resulted in the same clients visiting different providers attempting to find someone with stock. However, our tally sheet did not capture this type of dependence. Comparisons across the channels using the tally sheet data should be made with caution because data from the outreach and youth club providers could represent all clients seeking ECP services in the study areas during the 3-month period. However, while CBDAs and HSAs may be serving the majority of ECP clients, we cannot say our data contain all clients served by these channels. Lastly, our data are not verifiable with national-level data, but the perceptions of all the study populations were that ECP uptake increased in the areas where the strategy was implemented.

Despite these limitations, there were many strengths, including that our study populations reflect the health system structure by incorporating CHWs and outreach services yielding a more complete understanding of the context in which the strategy was implemented. Also, our mixed methods design allowed us to cross-check patterns emerging from various sources, and the consistency we found across study populations and data collection methods increases our confidence in the findings.^51^

## OPPORTUNITIES FOR IMPROVEMENT AND FURTHER SCALING UP

Based on our findings, the Malawi EC Strategy is likely increasing access to and uptake of ECPs, especially among youth and those in rural areas. We recommend that the MOH continue to raise awareness of the strategy among implementers, providers, and target end users and further scale up implementation. FP service delivery guidelines and sexual and reproductive health and rights policies should also be updated to reflect ECP strategy recommendations, including strengthening linkages to the concept of self-care. The MOH, with its partners, should develop an actionable scale-up plan for the implementation of the strategy across all districts in Malawi, with full coverage of the service through training of more providers at both the facility and, especially, the community level. To further ensure effectiveness of the strategy, commodity security is paramount so that clients can access their preferred number of EC packs, and strategies should be put in place to reduce the supply chain bottlenecks for ECPs. Once providers are trained, the MOH and its partners should intensify regular supportive supervision and follow-up of the trained providers to ensure correct information is provided to the clients, provider biases around repeat use of ECPs or adolescent sexual activity do not hamper access or uptake, and clients’ method choice is honored.

Based on our findings, the Malawi EC Strategy is likely increasing access to and uptake of ECPs, especially among youth and those in rural areas.

Our study highlights several areas for future research. First around equity, because not all who seek ECPs receive them, future research could explore potential differences in clients not receiving ECPs, for example, those less likely to be married, less educated, or poorer. Also, future research should be conducted with young people who are not being served to identify additional ways to further expand access. Also, our study was not designed to assess young people’s willingness to receive additional counseling messages in the youth-service channels. However, now that we documented these channels are reaching young people, future research should explore this question.

Finally, some providers reported hearing about instances of coercion in our study. While we cannot draw firm conclusions with our data, this is something worth exploring further as the strategy is expanded. Following guidance in the couples’ communication HIP, research should be conducted to ensure that interventions do not undermine women’s autonomy.^52^ Some example research questions include: To what extent are women’s and girls’ ECP use impacted by male partner coercion? What is the role of dual protection/condom use as we encourage male involvement? To what extent are men using ECPs to avoid using condoms, and what impact is this having on STIs? And how can programs reduce potential coercion? This may include training girls in assertiveness and boys in the need for consent. Then, these learnings, which are far more encompassing than ECPs, could be integrated into the adolescent package we described earlier and incorporated into future male involvement and demand creation activities.

While this is the first research conducted on Malawi’s EC strategy, it yielded actionable findings that can be applied within Malawi and globally. Given our encouraging results, we recommend continued provision of ECPs in the channels we studied, especially youth clubs and CBDAs, and expanding the strategy to other parts of the country. Policymakers in other countries should incorporate ECPs into their national FP programs, consider developing a similar strategy (or adapting existing policies to integrate these insights), and increase ECP access through novel, public-sector channels that meet the specific needs of youth—expanding method choice and paving the way for other contraceptive self-care options.

## Notes

**Peer Reviewed**

**First Published Online:** October 3, 2024.
